# Neuroprotective Effect of Bean Phosphatidylserine on TMT-Induced Memory Deficits in a Rat Model

**DOI:** 10.3390/ijms21144901

**Published:** 2020-07-11

**Authors:** Minsook Ye, Bong Hee Han, Jin Su Kim, Kyungsoo Kim, Insop Shim

**Affiliations:** 1 Department of Biomedicine & Health Sciences, College of Medicine, The Catholic University of Korea, Seoul 06591, Korea; jh486ms22@naver.com; 2 Department of Physiology, College of Medicine, Kyung Hee University, Seoul 02435, Korea; hanbh10@hanmail.net; 3 Division of RI-Convergence Research, Korea Institute of Radiological and Medical Sciences, Seoul 01812, Korea; kjs@kirams.re.kr

**Keywords:** neuroprotection, neurodegenerative disorder, choline acetyltransferase (ChAT), trimethyltin (TMT), bean phosphatidylserine (Bean-PS)

## Abstract

Background: Trimethyltin (TMT) is a potent neurotoxin affecting various regions of the central nervous system, including the neocortex, the cerebellum, and the hippocampus. Phosphatidylserine (PS) is a membrane phospholipid, which is vital to brain cells. We analyzed the neuroprotective effects of soybean-derived phosphatidylserine (Bean-PS) on cognitive function, changes in the central cholinergic systems, and neural activity in TMT-induced memory deficits in a rat model. Methods: The rats were randomly divided into an untreated normal group, a TMT group (injected with TMT + vehicle), and a group injected with TMT + Bean-PS. The rats were treated with 10% hexane (TMT group) or TMT + Bean-PS (50 mg·kg^−1^, oral administration (p.o.)) daily for 21 days, following a single injection of TMT (8.0 mg/kg, intraperitoneally (i.p.)). The cognitive function of Bean-PS was assessed using the Morris water maze (MWM) test and a passive avoidance task (PAT). The expression of acetylcholine transferase (ChAT) and acetylcholinesterase (AchE) in the hippocampus was assessed via immunohistochemistry. A positron emission tomography (PET) scan was used to measure the glucose uptake in the rat brain. Results: Treatment with Bean-PS enhanced memory function in the Morris water maze (MWM) test. Consistent with the behavioral results, treatment with Bean-PS diminished the damage to cholinergic cells in the hippocampus, in contrast to those of the TMT group. The TMT+Bean-PS group showed elevated glucose uptake in the frontal lobe of the rat brain. Conclusion: These results demonstrate that Bean-PS protects against TMT-induced learning and memory impairment. As such, Bean-PS represents a potential treatment for neurodegenerative disorders, such as Alzheimer’s disease.

## 1. Introduction

Trimethyltin (TMT) intoxication is regarded as an appropriate model of chronic neuronal degeneration associated with cognitive impairment, and is therefore useful in the study of Alzheimer’s disease (AD) [1,2]. The organotin trimethyltin chloride (TMT) is a neurotoxin that induces neuronal degeneration in the central nervous system (CNS) [3]. In particular, TMT injection leads to substantial damage of the hippocampus, which is implicated in memory [4]. Necrosis of hippocampal pyramidal cells and granule cells, produced by TMT, has been associated with the disruption of normal behavioral patterns [5], hippocampal physiological activity [6,7], and neurochemical markers of endogenous hippocampal neurotransmitters [7,8]. Rats exposed to TMT show behavioral, biochemical, and histological deficits [4]. For example, granule cells in the dentate gyrus, and cornu ammonis 1 (CA1) and cornu ammonis 3 (CA3) pyramidal cells, were significantly impregnated with TMT [9,10,11]. TMT intoxication attenuates hippocampal-dependent behavior in the Morris water maze [12] and the passive avoidance test [13]. TMT injection causes massive neuronal death, accompanied by enhanced hippocampal neurogenesis in the rat brain [14].

Several studies have shown that the cognitive dysfunction was associated with damaged cholinergic neurons in the brains of animal models of AD. Damage to the cholinergic system in the brain is closely associated with memory deficits. These anatomical and behavioral findings in TMT-intoxicated rats have been used to develop an attractive model of degenerative diseases such as AD [15]. AD has also been correlated with the loss of cholinergic neurons and decreased levels of acetylcholine (ACh) and choline acetyltransferase (ChAT). Lesions in these pathways lead to decreased ACh release, resulting in learning and memory dysfunction [16]. Current therapeutic measures are designed to increase levels of ACh in the brains of AD patients via suppression of acetylcholinesterase. Drugs, including the cholinesterase inhibitors, donepezil, galantamine, and rivastigmine, slow the breakdown of synaptic ACh, prolong its ability to stimulate post-synaptic receptors, and amplify the natural pattern of ACh release in the brain [17,18]. The drugs currently approved for the treatment of AD act by countering the acetylcholine deficits, leading to symptomatic relief, improved cognitive function, and enhanced acetylcholine levels in the brain.

Phosphatidylserine (PS) is the major anionic phospholipid found in the inner leaflet of eukaryotic cell membranes. PS-supplemented rodents showed enhanced memory, learning capacity, and other cognitive parameters [19]. In human studies, the efficacy of bovine-brain-derived PS (BC-PS) has been reported in patients with dementia [20]. Treatment with BC-PS improved memory function, especially delayed recall in the elderly with memory complaints. Although PS extracts from bovine cortex are known to be effective in improving memory function in humans and animals, alternative sources of PS are increasingly in demand [21]. It has been demonstrated that soybean-derived PS, one of the most promising alternatives, improved memory function in humans as much as BC-PS [22]. In addition, the treatment of rodents with BC-PS improved scopolamine-induced amnesia in the passive avoidance test, and cognitive disorders in senile subjects [23].

However, few studies have reported the effects of soybean-derived phosphatidylserine (Bean-PS) on cognitive improvement and its underlying mechanisms. The purpose of this study is to investigate whether Bean-PS prevents the neurodegeneration of the hippocampus, and the impairment of learning and memory, induced by TMT. The study demonstrated that Bean-PS improved cognitive function, and activated cholinergic systems in the hippocampus in addition to neural activation, in rats with TMT-induced cognitive deficits.

## 2. Results

### 2.1. Morris Water Maze Test

**Figure 1:**
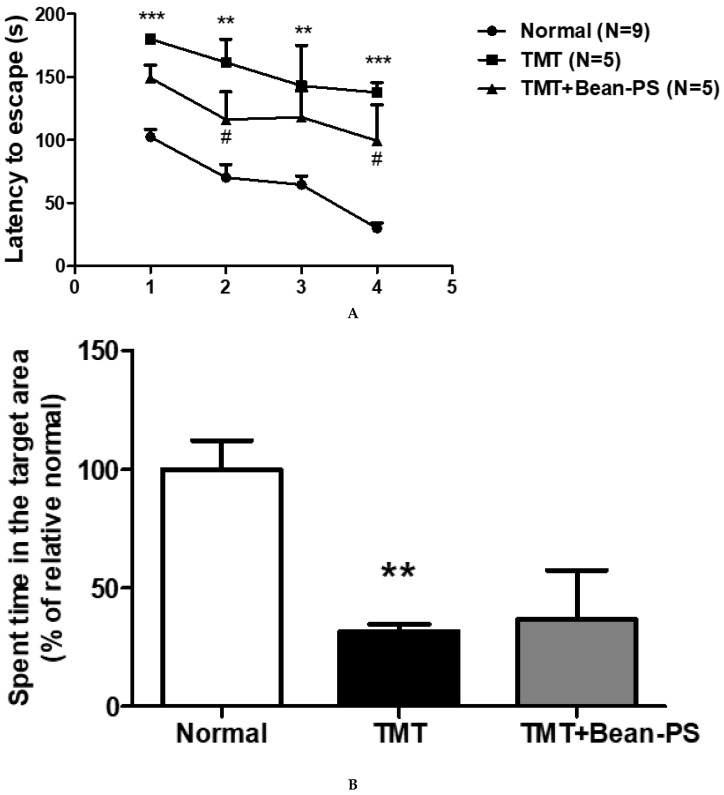
Effects of soy-bean-derived phosphatidylserine (Bean-PS) on spatial learning and memory dysfunction in trimethyltin (TMT)-induced rats. (**A**) The latency in escaping onto the hidden platform during the acquisition test. In the acquisition test, the task entailed three trials each day over 4 days. The values are presented as mean ± S.E.M. ** *p* < 0.01, *** *p* < 0.001 vs. normal group, # *p* < 0.05 vs. TMT group, respectively. (**B**)Retention was tested on Day 5. Results are expressed as means ± S.E.M. ** *p* < 0.01 vs. normal group. Normal group (*N* = 9); TMT group (*N* = 5); TMT-Bean-PS (*N* = 5).

Figure 1A represents the escape latency intervals recorded during successive training trials. In the acquisition trials, the TMT group showed deteriorated cognitive deficits compared with the normal group, reflected by the increased escape latency (*** *p* < 0.001, on Days 1 and 4, ** *p* < 0.01, on Days 2 and 3). The TMT + Bean-PS group demonstrated amelioration of spatial memory and learning ability relative to the TMT group starting from Day 2 (*p* < 0.05) and Day 4 (*p* < 0.05). In the retention test, the times spent on the platform varied significantly among the groups. The TMT group spent less time on the platform than the normal group (F_2, 18_ = 6.42, *p* < 0.05). The TMT + Bean-PS group did not affect the time spent in the platform area as seen in Figure 1B.

### 2.2. Passive Avoidance Test

**Figure 2:**
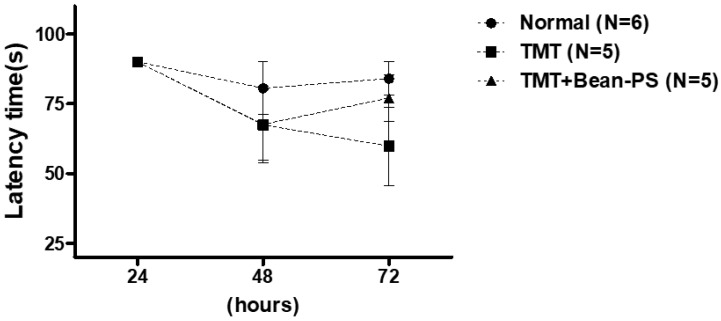
Effect of Bean-PS on escape latency into the dark phase of the retention test during the passive avoidance task. Each value is expressed ± S.E.M. Normal group (*N* = 6); TMT group (*N* = 5); TMT-Bean-PS (*N* = 5).

The passive avoidance test was conducted to determine the ability of working memory and learning. As shown in Figure 2, there was no significant difference among the three groups.

### 2.3. ChAT and AchE Immunoreactivity

**Figure 3:**
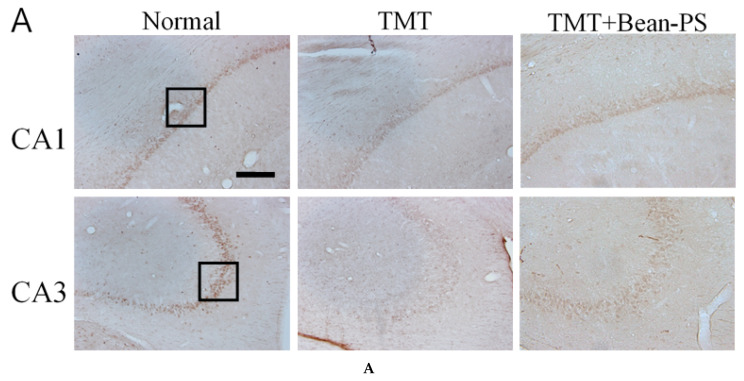

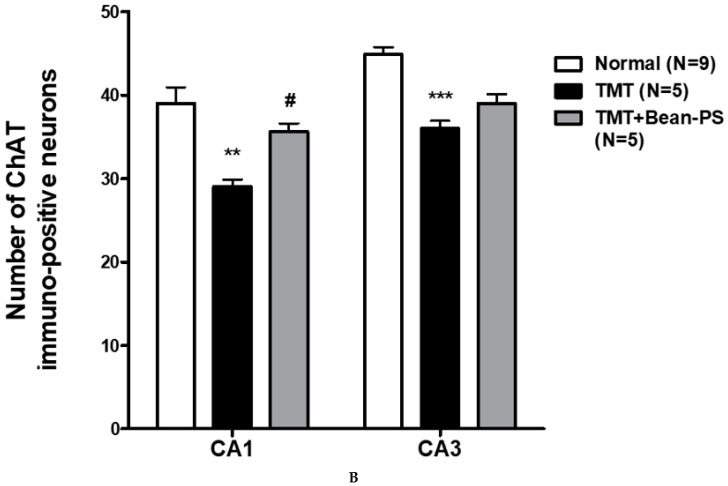
Effect of Bean-PS on the number of choline acetyltransferase (ChAT)-positive neurons in the hippocampus. Representative photographs and the number of positive neurons are indicated in (**A**) and (**B**). Black square represents region of CA1 and CA3 in the hippocampus and the scale bar represents 200 μm. Results are expressed as means ± S.E.M. *** *p* < 0.001, ** *p* < 0.01 vs. normal group, # *p* < 0.05 vs. TMT group. Normal group (*N* = 9); TMT group (*N* = 5); TMT-Bean-PS (*N* = 5).

The results of the evaluations of the ChAT-positive cells per section from the different hippocampal formations are shown in Figure 3. The ChAT activity in the hippocampus of the normal group was significantly higher than that of the TMT group. In particular, there were significant differences in the hippocampal CA1 (F_2, 18_ = 8.53, *p* < 0.01) and CA3 (F_2, 18_ = 22.94, *p* < 0.001).

**Figure 4:**
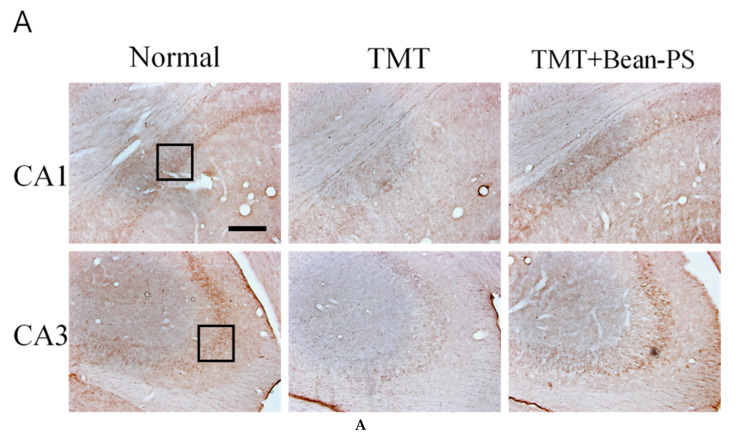

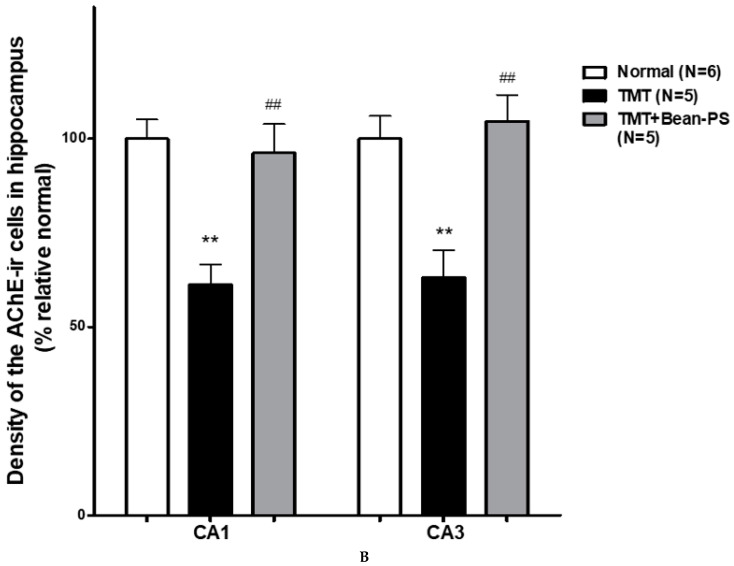
Effect of Bean-PS on the density of acetylcholinersterase (AchE)-immunostained nuclei in the hippocampus. Representative photographs and the density of AchE-immunostained nuclei are indicated in (**A**) and (**B**). Black square represents region of CA1 and CA3 in the hippocampus and the scale bar represents 200 μm. The values are presented as means ± S.E.M. ** *p* < 0.01 vs. normal group, ## *p* < 0.01 vs. TMT group, respectively. Normal group (*N* = 6); TMT group (*N* = 5); TMT-Bean-PS (*N* = 5).

The numbers of ChAT-positive cells in the TMT+Bean-PS group were higher than those in the TMT group, particularly in CA1 (*p* < 0.05). Immunoreactivity of ChAT in the TMT + Bean-PS group was significantly increased in CA1 (*p* < 0.05), as seen in Figure 3A,B. The TMT + Bean-PS group in the CA3 region showed no difference compared with that of the TMT group as seen in Figure 3A,B. The results of the evaluations of the acetylcholinesterase (AchE) immunoreactive cells per section from the different hippocampi are shown in Figure 4A,B. The AChE activity in the hippocampus of the TMT group was significantly lower than that of the normal group (*p* < 0.001). In particular, there were significant differences in both CA1 (F_2, 15_ = 12.07, *p* < 0.01) and CA3 (F_2, 15_ = 10.85, *p* < 0.01). However, the AChE reactivity in the TMT+Bean-PS group was higher than that in the TMT group, particularly in CA1 (*p* < 0.01) and CA3 (*p* < 0.01).

### 2.4. Brain Glucose Metabolism

**Figure 5:**
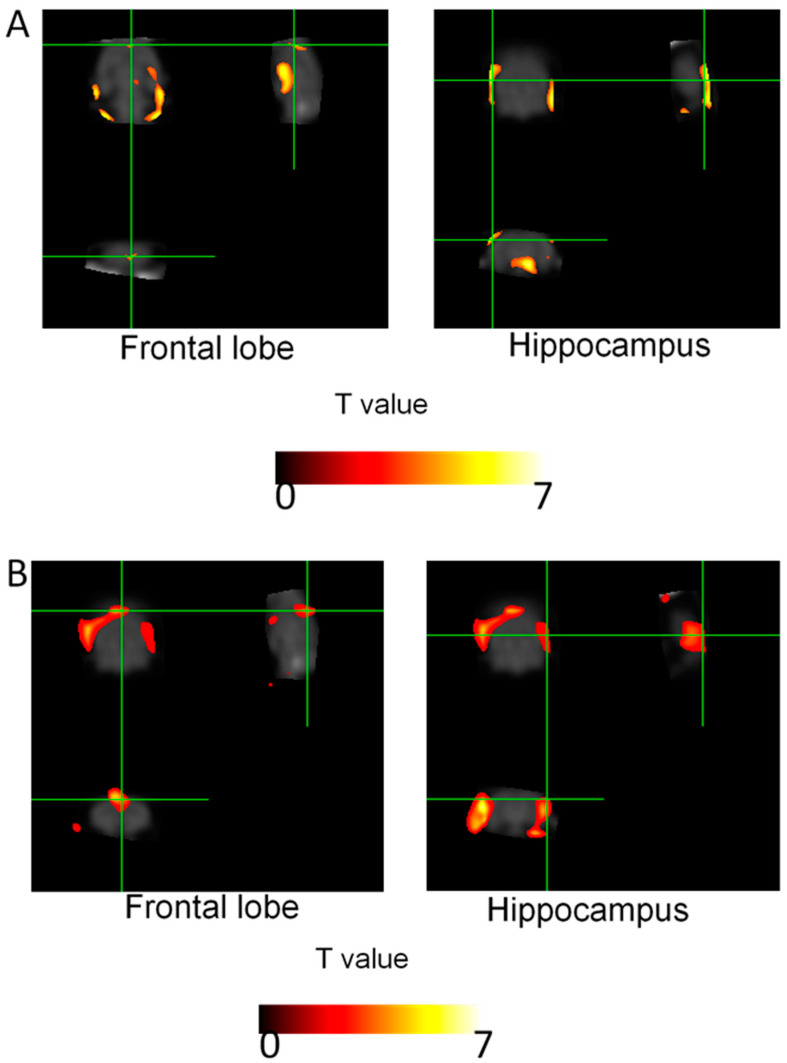
Brain regions showing regional glucose metabolism. (**A**) Brain regions where the regional ^18^F-fluodeoxyglucose (FDG) uptake in the TMT group (*N* = 3) was markedly lower than in the normal group (*N* = 3) (hippocampus and frontal lobe). (**B**) Brain region where reginal glucose uptake in the TMT + Bean-PS group (*N* = 3) was significantly higher than in the TMT group (frontal lobe and hippocampus). Green line: cross hair

Figure 5 and Table 1 show the results of ^18^F-fluorodeoxyglucose (FDG) uptake measurements from the different brain regions. The cerebral glucose activity of the TMT group was markedly reduced in the hippocampus and frontal lobe compared with that of the normal group in statistical parametric mapping (SPM) analysis of FDG-positron emission tomography (PET) (Figure 5A and Table 1A, *p* < 0.05). In addition, the activity of the TMT + Bean-PS group was significantly increased in the frontal lobe and hippocampus compared with the TMT group (Figure 5B and Table 1B, *p* < 0.05).

## 3. Discussion

AD is characterized by deterioration in memory, thinking, and the ability to carry out activities. An estimated 50 million people worldwide manifest dementia, and almost 10 million new cases are reported every year [24]. There is no established therapeutic agent currently available to treat dementia. Numerous new treatments are being investigated, in various stages of clinical trials. This study suggested that injection of TMT caused critical deficits in performance during tests of cognitive function, as well as causing corresponding signs of neurodegeneration, including decreased cholinergic neurons in the hippocampus. Our results showed that administration of Bean-PS reduced the TMT-induced learning and memory deficits in the Morris water maze, and suppressed TMT-induced reduction in ChAT and AchE in the hippocampus.

TMT exposure triggers severe behavioral and cognitive defects in both humans [25] and experimental animals [26]. TMT injection induced damage to the hippocampal pyramidal neurons, and also in the associated areas in rats [26,27,28,29,30,31,32,33]. Furthermore, previous studies have reported that TMT injection increases the risk of neuronal cell death, via possible excitotoxicity, intracellular calcium overload, and mitochondrial damage [34]. Several behavioral studies have suggested that the performance of TMT-induced rats is poor in memory and learning tasks [35,36]. The Morris water maze test has been used to test permanent spatial learning ability and reference memory, and utilized to evaluate cognitive-enhancing agents for the treatment of neurocognitive disorders [37,38].

Our study indicated that spatial memory impairment was ameliorated in the TMT+Bean-PS groups during the training days in contrast with that of the TMT group. In addition, the data demonstrated that Bean-PS preserved the TMT-induced reduction in spatial retention. These results suggested that Bean-PS improved learning and memory deficits in TMT-intoxicated rats used as experimental models of neurodegeneration for the study of Alzheimer-like diseases [15,39].

The animal model used in this study clearly demonstrates the functional significance of hippocampal neurodegeneration induced by TMT. The cholinergic system is involved in information processing associated with hippocampal learning and memory [40]. The hippocampus carries information derived from the related regions of the brain that are involved in learning and memory [41], and any damage to the cholinergic system may result in altered behavioral responses [42]. In particular, the loss of cholinergic function has been associated with a decline in cognition during aging and in AD [43]. Thus, the expression of AchE and ChAT in the hippocampus and its relation to TMT-induced cognitive impairment in rats was examined. Bean-PS also continuously improved the activity of cholinergic neurons in the hippocampus, which eventually restored the cholinergic pathway [44].

Based on the cholinergic hypothesis, patients with senile dementia show a selective and irreversible lack of cholinergic function in the brain [45]. Therefore, in patients with AD, treatment with cholinesterase inhibitors and ChAT activators may compensate for decreased ACh levels.

It is quite probable that the observed improvement in spatial learning deficits following the treatment with Bean-PS of rats was related to the enhanced release of ACh. According to the results from the Morris water maze (MWM), exposure to Bean-PS ameliorated the TMT-induced deficits in learning and memory. In addition, treatment with Bean-PS decreased cell loss, increased central cholinergic function, and prevented degeneration of cholinergic neurons mediating cognitive processes [46].

Our previous study reported that treatment with Bean-PS dissolved in medium-chain triglyceride (MCT) improved cognitive function and enhanced the neural activity in rats with TMT-induced learning and memory deficits. The active biological ingredient in the resulting extract is affected by the solvents [47]. Therefore, we investigated how the activity of Bean-PS in 10% hexane solvent affected TMT-induced memory deficits in rats.

The present study analyzed the effect of 10% hexane solvent on Bean-PS-attenuated TMT-induced cognitive defects in the Morris water maze, and found a protective effect in contrast to TMT-induced reduction in ChAT and AchE in the hippocampal areas.

Exposure to Bean-PS resulted in upregulation of glucose uptake in the hippocampus and frontal lobe. Administration of Bean-PS may have robust therapeutic potential as a treatment for neurodegenerative disorders, such as AD.

## 4. Material and Methods

### 4.1. Animals and Experimental Design

Male Sprague-Dawley rats weighing 250–580 g each were purchased from Samtaco Animal Corp. All rats were housed individually in a room at 23 °C (room temperature) under an alternating 12 h light/dark cycle. The rats were fed a commercial diet and provided with tap water ad libitum throughout the study. Food and water were made accessible ad libitum. This experiment was conducted in accordance with the National Institutes of Health Guide for the Care and Use of Laboratory Animals, revised in 1996, and was approved by the Institutional Animal Care and Use Committee of Kyung Hee University (KHUAP(SE)-18-073, 05/30/2018). In this study, the rats were randomly assigned to three groups: untreated, naïve (Normal, *n* = 9); TMT injected with vehicle (TMT, *n* = 5); and TMT injected along with 50 mg/kg^−1^ Bean-PS (Bean-PS, *n* = 5). The rats were injected intraperitoneally (i.p.) with TMT (8.0 mg/kg, body weight) dissolved in 0.9% saline and then returned to their home cages. The TMT+Bean-PS mixture (50 mg/kg, oral administration (p.o.)) was dissolved in 10% hexane and orally administered for two weeks after TMT-induced neurodegeneration.

### 4.2. Drug Treatment

PS was produced from soy lecithin by enzymatic transphophatidylation, and comprised a mixture of 90% phosphatidylserine (PS), 2% phosphatidylcholine (PC), and 6% phosphatidic acid (PA). The Bean-PS contained palmitic (17.7%), palmitoleic (1.3%), stearic (1.3%), oleic (14.4%), limoleic (61.2%), linolenic (1.4%), eicosapentaenoic acid (EPA), and other fatty acids (9.4%). PS and 10% hexane, used as a solvent for PS, were provided by Doosan Co. Glonet BG (Youngin, Korea) and the PS was stored in a freezer (−60 °C).

### 4.3. Morris Water Maze (MWM)

From the 17th day after the treatment with Bean-PS, the Morris water maze test was performed for 5 days. The black, plastic pool used in the Morris water maze test measured 200 cm in diameter and was filled to a depth of 35 cm with clear water maintained at 23 ± 2 °C. It was inserted in a submerged platform using external cues around the pool and within the behavioral room. For behavioral analysis, a personal computer was used to assort a charge coupled device (CCD). A probe test was run after the last trial on Day 4. In the experiment, the rats were exposed to an acquisition trial on each of 4 consecutive days. On Day 5, they were also trained in retention tests. The rats were allowed to search for the platform for 180 s during the acquisition test. On Day 5 of the retention test, the rats underwent a 1 min probe trial, in which the platform was eliminated from the pool. Probe trials were run at 1 min intervals. The animals in the performance test of each water maze trial were evaluated with a personal computer during the behavioral analysis (S-mart program, Barcelona, Spain).

### 4.4. Passive Avoidance Task (PAT)

A passive avoidance task was performed after 21 days of the treatment with Bean-PS. A test chamber was composed of two platforms, one light (white partition, 20 cm × 20 cm × 30 cm) and the other dark (black partition, 20 cm × 20 cm × 30 cm). A guillotine door opening (6 cm × 6 cm) was made on the floor in the center of the divider between the two partitions. Stainless steel bars, of diameter approximately 5 mm, were spaced at about 1 cm apart. Electric shocks (5 V, 0.5 mA, 10 s) were transferred to the grid on the floor of the dark compartment. All rats were again located on the platform, and the escape latency was recorded in the retention test.

### 4.5. Immunohistochemical Staining

After completion of the ^18^F fluorodeoxyglucose (FDG), rats were anesthetized with sodium pentobarbital (100 mg·kg^−1^, intraperitoneally). Rats were perfused with saline solution, followed by 4% paraformaldehyde (PFA). The brain was anatomized from the skull, post-fixed in PFA overnight, and stored in a 30% sucrose in PBS until it subsided. The brain was embedded and serially sectioned on a cryostat (Leica Microsystem Co., Ltd., Wetzlar, Germany) at 30 μm thickness in the coronal plane and the sections were collected in phosphate buffered saline (PBS). The primary antibodies against the following specific antigens were used: ChAT and AchE. The primary antibody was diluted from the concentrate with the blocking solution (0.2% phosphate buffered saline with tween 20 (PBST), 2% blocking serum in PBST). The primary antibody was infused into the brain sections for 72 h at 4 °C. After several rinses in PBST, the sections were incubated with secondary biotinylated antibodies against rabbit immunoglobulin G (IgG) or sheet IgG (Vector Laboratories, Burlingame, CA, USA) for 2 h. After washing with PBST, the sections were incubated with an appropriate biotinylated secondary antibody and processed with an avidin–biotin complex kit (Vectastain ABC kit; Vector Laboratories, Burlingame, CA, USA). The staining was conducted using 0.05% 3,3′-Diaminobenzidine (DAB) in the presence of 0.003% H_2_O_2_ in 0.1 M PB. After rinsing with 0.1 M PB, the stained tissue sections were mounted on the slide. The images were captured using a DP2-BSW imaging system (Olympus, CA, USA) and processed using Adobe Photoshop Cells. Those testing positive for ChAT and AChE were counted on a grid that was placed on CA1 and CA3 in the hippocampus area. The number of cells was counted at a magnification of 100×, using a rectangular microscopic grid measuring 200 × 200 µm^2^. The cells were counted in 3 sections per rat within the hippocampus.

### 4.6. Image Processing and Analysis

After completion of the behavioral test, animals underwent prior fasting over 12–15 h and bedding was changed during the fasting period to avoid ingestion of bedding. The animals were treated with 500 mCi/100 g 18F-FDG via tail intravenous insjsmsjection, and the animals inhaled 2% isoflurane in 100% oxygen (Forane solution; ChoongWae Pharma, Korea) until the positron emission tomography (PET) scan. A transverse resolution of <1.8 mm was used at the center [48]. The emission data were acquired at an energy window setting (350–650 keV, 30 min). The acquired data in the emission list mode were arranged into three-dimensional (3D) sinograms and reconstructed using 3D Reprojection (3DRP) methods. To distinguish the cerebral glucose metabolism, between TMT + Bean-PS and TMT group datasets, a voxel-based statistical analysis was performed using SPM. In summary, the area of the brain was masked using the rectangular method. The PET data were spatially reconstructed onto a rat brain template, smoothed using a 3 mm isotropic Gaussian kernel, and counted. A Statistical Parametric Mapping 8 program was used in the voxel-wise t-test between the TMT + Bean-PS and TMT group datasets (*p* < 0.05, K > 50).

### 4.7. Statistical Analysis

Results were assessed using one-way or two-way analysis of variance (ANOVA), and repeated measures of ANOVA, followed by Tukey’s post hoc test, using SPSS 15.0 for Windows, for statistical analysis, for multi-group comparisons, and Student’s *t* test for single comparisons, using Prism 5.01 software (Graphpad Software Inc., San Diego, CA, USA). In all statistical assessments, *p* < 0.05 was considered to be statistically significant. Results are expressed as the mean ± standard error of the mean.

## 5. Conclusions

Treatment with Bean-PS attenuated the TMT-induced memory impairment in the MWM test. Furthermore, the Bean-PS improved cognitive function, and generated a neuroprotective effect by increasing the expression of cholinergic neurons and glucose metabolism. Thus, Bean-PS is a potential agent that can be used to prevent and protect against neurodegenerative diseases such as dementia and AD. It may represent a good therapeutic agent for of the management of AD.

## Figures and Tables

**Table 1:** The changes of Z values in the hippocampus and frontal lobe. The results of voxel-wise comparison between the TMT + Bean-PS and TMT group datasets are shown (Table 1A). In the statistical parametric mapping (SPM) analysis, the cerebral glucose uptake of TMT+Bean-PS datasets was significantly increased in the hippocampus and frontal lobe compared with that of the TMT group (Table 1B).

**A**		
**Brain Area**	**Coordinates (x, y, z)**	**Z Value**
Frontal lobe	(8, 1, 10)	1.95
Hippocampus (Right)	(9, −1, 11)	1.69
Hippocampus (Left)	(−9, −1, 10)	1.66
**B**		
**Brain Area**	**Coordinates (x, y, z)**	**Z Value**
Frontal lobe	(2, 4, 8)	3.24
Hippocampus (Right)	(7, −3, 3)	4.57
Hippocampus (Left)	(−5, −5, 1)	3.3
